# Influence of farnesoid X receptor (*FXR*) on lipid metabolism in calf hepatocytes exposed to high fatty acid levels

**DOI:** 10.3389/fvets.2026.1801528

**Published:** 2026-04-30

**Authors:** Bin Jia, Yan Tian, Changhong Gao, Yaqi Chang, Zexin Zhang, Yuxi Song, Cheng Xia, Yongli Qu, Wei Yang

**Affiliations:** 1 Heilongjiang Provincial Key Laboratory of Prevention and Control of Bovine Diseases, College of Animal Science and Veterinary Medicine, Heilongjiang Bayi Agricultural University, Daqing, China; 2 College of Veterinary Medicine, Yunnan Agricultural University, Kunming, China

**Keywords:** bile acid, dairy cow, fatty liver, FXR, lipid accumulation

## Abstract

**Introduction:**

Fatty liver is a common metabolic disease in dairy cows during early postpartum period, which is characterized by excessive hepatic triacylglycerol (TAG) accumulation. However, the mechanisms of bile acid (BA) metabolism in dairy cows experiencing fatty liver remain poorly elucidated. The farnesoid X receptor (*FXR*) plays a critical role in the regulation of BA homeostasis. Consequently, the aim of this study was to investigate the effect of *FXR*-mediated BA metabolism following stimulation with high concentrations of free fatty acids (FFA).

**Methods:**

*In vivo*, liver tissue from healthy control cows (*n* = 6; with hepatic TAG < 1%) and fatty liver cows (*n* = 6; with hepatic TAG > 2%) were used to evaluate the factors related to BA metabolism. *In vitro*, hepatocytes isolated from three healthy female calves were exposed to either 1.2 m*M* FFAs or kept as controls to simulate metabolic distress. Subsequently, hepatocytes were treated with either 5 µ*M* of the *FXR* activator GW4064 or 5 µ*M* of the *FXR* inhibitor (Z)-guggulsterone with or without the addition of 1.2 m*M* FFAs.

**Results:**

Our findings indicate that both *in vivo* and *in vitro* exposure to FFAs was associated with increased mRNA and protein abundance of bile acid synthesis-related factors (*CYP7A1*, *CYP8B1*) and mRNA expression of *CYP7B1*. Conversely, the expression of BA synthesis-related factors (*FXR*, *CYP27A1*) and BA transporters (*ABCC2*, *ABCB11*) were diminished in fatty liver cows compared to controls. Furthermore, compared to the control group, fatty acid synthesis-related factors (*SREBF1*, *ACC1*, *FASN*), a mitochondrial dysfunction marker (*VDAC1*), and oxidative stress indicators (ROS, H_2_O_2_) were upregulated in the FFA group. Additionally, cholesterol synthesis-related genes (*SREBF2*, *HMGCR*) were lower in the FFA group compared to the control group. Notably, compared to the FFA group, the GW4064 + FFA group showed reduced expression of *CYP7A1*, *CYP8B1*, *CYP27A1*, *CYP7B1*, *SREBF1*, *ACC1*, *FASN*, and *VDAC1*, along with decreased TAG, ROS, and H_2_O_2_ in hepatocytes. Conversely, the expression of *FXR*, *SREBF2*, *HMGCR*, *ABCC2*, and *ABCB11* was higher in the GW4064 + FFA group compared to the FFA group. Furthermore, the application of the *FXR* inhibitor (Z)-guggulsterone yielded results that were contrary those observed with GW4064.

**Discussion:**

Overall, our data suggest that FXR activation by GW4064 effectively attenuated high FFA-induced BA and lipid accumulation in calf hepatocytes, which ultimately alleviated hepatocyte oxidative damage.

## 1 Introduction

Fatty liver is a common metabolic disease in high-yielding dairy cows (1, 2). During the transition period, dairy cows experience a state of negative energy balance (NEB) due to reduced dry matter intake and increased energy requirements to maintain lactation (3). NEB leads to fat mobilization, resulting in elevated circulating free fatty acid (FFA) levels, which in turn causes hepatic triacylglycerol (TAG) accumulation and fatty liver development (4, 5). Severe fatty liver reduces the production performance of dairy cows and is often accompanied by other diseases (6, 7). Therefore, it is particularly important to identify effective therapeutic targets for fatty liver.

Hepatic steatosis in dairy cows is primarily attributed to dysregulation of fatty acid oxidation, TAG synthesis, and export from very low-density lipoprotein (VLDL) (8). As the main pathway of endogenous TAG transport in the liver, the synthesis and secretion of VLDL depend on the availability of cholesterol and TAG (9). In high-yielding dairy cows, a significant reduction in liver cholesterol concentrations during early lactation is part of the typical physiological changes associated with fatty liver (9, 10).

Bile acid (BA) is synthesized from cholesterol in the liver, secreted into bile as the main solute, and plays a role in promoting lipid absorption in the intestine (11, 12). Cholesterol is converted to BA via two main routes: the classical pathway, catalyzed by cholesterol 7α-hydroxylase (*CYP7A1*) and sterol 12α-hydroxylase (*CYP8B1*) (13), yielding cholic acid (CA), and the alternative pathway, catalyzed by sterol-27α-hydroxylase (*CYP27A1*) and *CYP7B1*, producing chenodeoxycholic acid (CDCA) (14). BA homeostasis is tightly regulated by various factors, including BA receptors, transporters, and proteins involved in enterohepatic circulation (15–17). However, the regulatory mechanisms of BA homeostasis in the liver of dairy cows with fatty liver remain unknown.

Farnesoid X receptor (*FXR*) is a key receptor regulating BA homeostasis *in vivo*, and it is highly expressed in the liver and intestine (18). As a key regulator of BA synthesis, metabolism, and transport, *FXR* plays a significant role in cholestatic liver diseases in humans (19). *FXR*-mediated activation of small heterodimer partner (SHP) represses *CYP7A1* expression, thereby inhibiting BA synthesis and uptake. It also represses the expression of sterol regulatory element binding transcription factor 1 (*SREBF1*) (20, 21). In mice, deficiency of *FXR* results in increased liver steatosis (22). However, it remains unclear how *FXR* regulates BA and lipid synthesis in the liver of dairy cows, particularly during NEB. Therefore, this investigation aims to elucidate the role of *FXR* in BA and lipid metabolism in calf primary hepatocytes.

## 2 Materials and methods

### 2.1 Animal ethics

The animal use protocol complied with the guidelines set forth by the Ethics Committee for the Use and Care of Animals, Heilongjiang Bayi Agricultural University (approval No. SMKXJSXY2022003).

### 2.2 Liver tissue collection

All cows with a similar number of lactations (range = 2 to 4) and days in milk (DIM; range = 8 to 12) were screened for this study from a commercial dairy farm (Heilongjiang Province, China). Liver biopsies were collected as described previously (9). Liver TAG was analyzed using an enzymatic assay kit (Applygen Technologies, Inc., Beijing, China) in accordance with the manufacturer’s instructions.

Cows with liver TAG content (expressed as the ratio of triglyceride weight to wet liver weight) below 1% were classified as healthy controls, and those with liver TAG content greater than 2% were classified as having fatty liver (1). Subsequently, six dairy cows with fatty liver and six healthy control cows were selected for subsequent experiments.

### 2.3 Isolation and culture of primary calf hepatocytes

In the present study, a total of three healthy newborn female Holstein calves (body weight: 42–53 kg; 1 d old) were purchased from a cow dairy farm (Daqing, Heilongjiang Province, China) and housed in a temperature-controlled environment.

Primary calf hepatocytes were isolated using a modified two-step collagenase perfusion method as described previously (9). Briefly, the caudate liver lobe was surgically resected from the liver. Blood was removed from the surface of the caudate lobe by rinsing with perfusion solution A (140 m*M* NaCl, 6.7 m*M* KCl, 10 m*M* HEPES, 2.5 m*M* glucose, and 0.5 m*M* EDTA, pH 7.4; 37 °C, 50 mL/min for 10–15 min).

Then, perfusion solution B (140 m*M* NaCl, 6.7 m*M* KCl, 30 m*M* HEPES, 2.5 m*M* glucose, and 5 m*M* CaCl_2_, pH 7.4; 37 °C, 50 mL/min for 5 min) was used to perfuse the caudate lobe until the liquid became clear. Subsequently, the liver was perfused with digestive fluid (0.1 g of collagenase IV dissolved in 0.5 L of perfusion solution B, pH 7.4; 20 mL/min for 15 min) to dissociate the liver tissue structure until the liquid became turbid.

Digestion was terminated with precooled (4 °C) fetal calf serum (FBS, Hyclone Laboratories, UT, USA). The liver was cut into small pieces, and the hepatic parenchyma was filtered sequentially through 100-mesh (150 μm) and 200-mesh (75 μm) cell sieves.

The obtained hepatocytes were washed twice with RPMI-1640 basal medium (Hyclone Laboratories, UT, USA) and centrifuged for 5 min at 500 × g at 4 °C. Primary hepatocytes (1 × 10^6^ cells/mL) were seeded into a 6-well tissue culture plate (2 mL per well) in adherent medium (RPMI-1640 basal medium supplemented with 10% FBS, 10^−6^ m*M* of insulin, 10^−6^ m*M* of dexamethasone, 10 μg/mL of vitamin C) and incubated for 4 h at 37 °C in 5% CO_2_.

The growth medium was then replaced every 24 h (RPMI-1640 basal medium supplemented with 10% FBS, 100 U/mL penicillin, and 100 μg/mL streptomycin).

### 2.4 FFA, GW4064, and (Z)-guggulsterone preparation

The concentration of free fatty acids (FFA) used in this study was selected according to hematological criteria of dairy cows with fatty liver (23, 24).

Briefly, to prepare the FFA stock, individual fatty acids were diluted in potassium hydroxide solution (0.1 *M*) and dissolved at 60 °C (including c9-18:1, 18:2, 16:0, 18:0, and c9-16:1 at 43.5, 4.9, 31.9, 14.4, and 5.3% of total fatty acids, respectively). Subsequently, the pH of the FFA solution was adjusted to 7.6 with hydrochloric acid (1 *M*).

The final concentration of the stock solution was 60 m*M*. After FFA preparation, the stock solution was aliquoted and stored at −20 °C until use.

GW4064 (HY-50108; Med Chem Express Co., Ltd., NJ, USA) was dissolved in dimethyl sulfoxide (DMSO) to achieve a final concentration of 5 m*M*. Similarly, (Z)-guggulsterone (HY-110066; MedChemExpress Co., Ltd., NJ, USA) was dissolved in dimethyl sulfoxide (DMSO) to a final concentration of 10 m*M*.

### 2.5 Activation and inhibition of FXR

Before the addition of *FXR* activator GW4064, hepatocytes were cultured in 6-well plates (1 × 10^6^ cells/mL, 2 mL per well) in growth medium for 44 h and starved for 12 h in serum-free RPMI-1640 basal medium.

Subsequently, the hepatocytes (*n* = 9 replicates per group) were divided into four groups: control, FFA, GW4064, and GW4064 + 1.2 m*M* free fatty acids (GW4064 + FFA).

In the control and FFA groups, the hepatocytes were maintained in RPMI-1640 basal medium containing 2% BSA and treated with or without 1.2 m*M* FFAs for 12 h. In the GW4064 and GW4064 + FFA groups, 5 μ*M* of *FXR* activator GW4064 was used for 12 h before incubation with or without FFAs.

To gain better insights into the role of *FXR* in dairy cows with fatty liver *in vitro*, hepatocytes were treated with the *FXR* inhibitor (Z)-guggulsterone at a concentration of 5 μ*M* for 12 h before incubation with or without 1.2 m*M* FFAs. The cells (*n* = 9 replicates per group) were divided into four groups: control, FFA, (Z)-guggulsterone, and (Z)-guggulsterone + FFA.

### 2.6 mRNA extraction and RT-qPCR

Total mRNA was extracted from hepatocytes (*n* = 9 replicates per group) using TRIzol (Invitrogen Corporation, Carlsbad, CA, USA) following the manufacturer’s protocol. Total RNA (1 μg) was transcribed into cDNA using Reverse Transcriptase M-MLV (RNase H–) (RR047A, TaKaRa Biotechnology Co., Ltd., Japan) following the manufacturer’s protocol.

The mRNA abundance was determined using FastStart Universal SYBR Green Master (ROX) (04913914001, Roche, Norwalk, CT, USA) with the Bio-Rad iCycler iQ™ Real-Time PCR Detection System (Bio-Rad Laboratories Inc., Hercules, CA, USA).

The qRT-PCR reaction system consisted of 10 μL of FastStart Universal SYBR Green Master, 2 μL of cDNA, 1 μ*M* of each primer, and 6 μL of RNase-free distilled H_2_O to achieve a final volume of 20 μL.

Evaluated target genes were *SREBF1*, fatty acid synthase (*FASN*), acetyl-CoA carboxylase 1 (*ACC1*), sterol regulatory element-binding protein 2 (*SREBF2*), 3-hydroxy-3-methyl-glutaryl coenzyme A reductase (*HMGCR*), *CYP7A1*, *CYP7B1*, *CYP8B1*, cholesterol 27α-hydroxylase (*CYP27A1*), multidrug resistance-associated protein 2 (*ABCC2*), bile salt export pump (*ABCB11*), and *FXR*.

Relative mRNA abundance was normalized to the geometric mean of glyceraldehyde-3-phosphate dehydrogenase (*GAPDH*) and *β*-actin (*ACTB*). *GAPDH* and *ACTB* maintained stable expression under the conditions of FFA, GW4064, and (Z)-guggulsterone, as analyzed by BestKeeper (25).

Target gene mRNA abundance was calculated using the 2^–ΔΔCT^ method. Gene primers are shown in Table 1.

**Table 1:** Sequences of primers used for real-time PCR amplification.

Gene	Primers (5′–3′)
*SREBF2*	Forward: GACTGATGCCAAGATGCACA Reverse: CCCTTCAGGAGTTTGCTCTT
*HMGCR*	Forward: ACCCATGAGCGAGGTGTATC Reverse: TAGTGCTGGCCACAAGACAG
*CYP7A1*	Forward: GCCCGTGCTAGACAGTATCATCAAG Reverse: CGTCCTGGAGGTGCAAAGTGAAG
*CYP7B1*	Forward: TGCTTTCACCTGGCTGCTGTTAC Reverse: GGAGGCGGATTTGTTACCACTGAG
*CYP27A1*	Forward: ACACGACATCCAACACGCTGAC Reverse: CACCACACCCACCACTTCCTTATG
*CYP8B1*	Forward: GCAGAGGAAGCTAGACTTTGTGGAG Reverse: GCTTGGTGCTGGCTGAGTGTATC
*FXR*	Forward: TGCCTTTGTAAGCCTCAGTTTCACC Reverse: TCCTTTCCTCACCACCCACTTCC
*ABCB11*	Forward: GGCACTGGACAATGAGAGCGAAG Reverse: GATAGGCGATGAGCGACAGAGATG
*ABCC2*	Forward: CTGTGTCAGGCTTGTCTGTTATCCG Reverse: TTCTGGTTGGTGTCAATCGCTGTC
*FASN*	Forward: ACAGCCTCTTCCTGTTTGACG Reverse: CTCTGCACGATCAGCTCGAC
*ACC1*	Forward: GCTGAATATCCTCACGGAGCT Reverse: CGACGTTTCGGACAAGATGAGT
*SREBF1*	Forward: GCAGCCCATTCATCAGCCAGACC Reverse: CGACACCACCAGCATCAACCACG
*ACTB*	Forward: GCTAACAGTCCGCCTAGAAGCA Reverse: GTCATCACCATCGGCAATGAG
*GAPDH*	Forward: GTCTTCACTACCATGGAGAAGG Reverse: TCATGGATGACCTTGGCCAG

### 2.7 Protein extraction and Western blotting

Total protein from hepatocytes (*n* = 9 replicates per group) was lysed using RIPA buffer (Beyotime Biotechnology, Jiangsu, China) with protease inhibitors. The supernatant was aspirated, and protein concentration was measured using the BCA protein assay kit (Beyotime, China).

Aliquots of total protein (30 μg/lane) were subjected to 8% sodium dodecyl sulfate–polyacrylamide gel electrophoresis, and protein was transferred onto a 0.45-μm polyvinylidene difluoride membrane (Millipore Corp., Billerica, MA, USA).

Then, the membrane was saturated with 5% non-fat milk and incubated with primary antibodies against *SREBF1* (1:1,000, NB100–2215; Novus Biologicals, Littleton, CO, USA), *FASN* (1:1,000, C2065; Cell Signaling, Danvers, MA, USA), *ACC1* (1:2,000, Abcam, ab45174, Cambridge, MA, USA), *SREBF2* (1:500, ab30682 Abcam, Cambridge, MA, USA), *HMGCR* (1:1,000, A1633, ABclonal, China), *CYP7A1* (1:1,000, A10615, ABclonal, China), *CYP8B1*(1:1,000, K109819P, Solarbio, China), *CYP27A1*(1:1,000, A1982, ABclonal, China), *FXR* (1:5,000, 25,055-1-AP, proterntech, China), *VDAC1* (1:1,000, ab15895, Abcam, Cambridge, MA, USA), and *β*-actin (1:1,000, sc-47778; Santa Cruz Biotechnology, CA, USA) overnight at 4 °C.

The membrane was then washed with TBST and incubated with HRP-conjugated anti-rabbit or anti-mouse antibody (1:5,000; Beyotime Biotechnology, Beijing, China) for 40 min at room temperature.

The immune response signal was observed using a ProteinSimple imager (ProteinSimple, San Jose, CA, USA) with an enhanced chemiluminescence solution (Beyotime Biotechnology, China).

Band intensity analyses of immunoblots were carried out using Image Lab software (Bio-Rad Laboratories Inc., Hercules, CA, USA).

Based on the stable expression of *β*-actin in Western blot assays as a loading control under FFAs, GW4064, and (Z)-guggulsterone conditions, target protein abundance was normalized to β-actin abundance.

### 2.8 Detection of TAG content and H_2_O_2_ content

The content of TAG in liver tissue and primary hepatocytes was determined using an enzymatic assay kit (Applygen Technologies, Inc., Beijing, China) in accordance with the manufacturer’s instructions.

The TAG content in liver tissue was expressed as the ratio of triglyceride weight to wet liver weight.

For primary hepatocytes, the cells were also used to determine protein concentration via the BCA protein assay kit (Beyotime Biotechnology, China) to normalize TAG content.

### 2.9 Lipid droplet fluorescence detection

Approximately 5,000 cells per well were seeded into a 12-well culture plate. A 1-mL cell suspension was added to each well and treated with or without GW4064 or (Z)-guggulsterone and then incubated with or without 1.2 m*M* FFA.

Subsequently, the cells were washed twice with PBS and fixed for 30 min with 4% paraformaldehyde. After washing with PBS three times, BODIPY 493/503 (Invitrogen Corporation, Carlsbad, CA, USA) dye was added for 30 min in the dark.

Cells were washed twice with PBS and incubated with Hoechst 33342 dye (Beyotime Biotechnology, China) for 8 min before fluorescence microscopy (IX73, Olympus, Tokyo, Japan).

### 2.10 ROS detection

Reactive oxygen species (ROS) levels were determined using an assay kit (S0033S, Beyotime). A total of 1 × 10^5^ cells were incubated with 10 μ*M* carboxy-2′,7′-dichloro-dihydro-fluorescein diacetate probe in PBS for 15 min at 37 °C.

Fluorescence was measured at 488 nm (excitation) and 525 nm (emission) using a Beckman CytoFLEX flow cytometer (Beckman Coulter).

### 2.11 Statistical analysis

All data were analyzed using SPSS 22.0 (SPSS Inc., Chicago, IL, USA) or Prism 7 statistical software. The results are reported as mean ± standard error of the mean.

Data obtained regarding liver tissue were compared using a two-tailed unpaired Student’s *t*-test. Data from calf hepatocyte treatment comparisons were assessed by one-way ANOVA, and multiplicity for each experiment was adjusted with the Bonferroni procedure to control for type I error rate at 0.05.

## 3 Results

### 3.1 Bile acid metabolism-related factors in healthy or fatty liver cows

**Figure 1:**
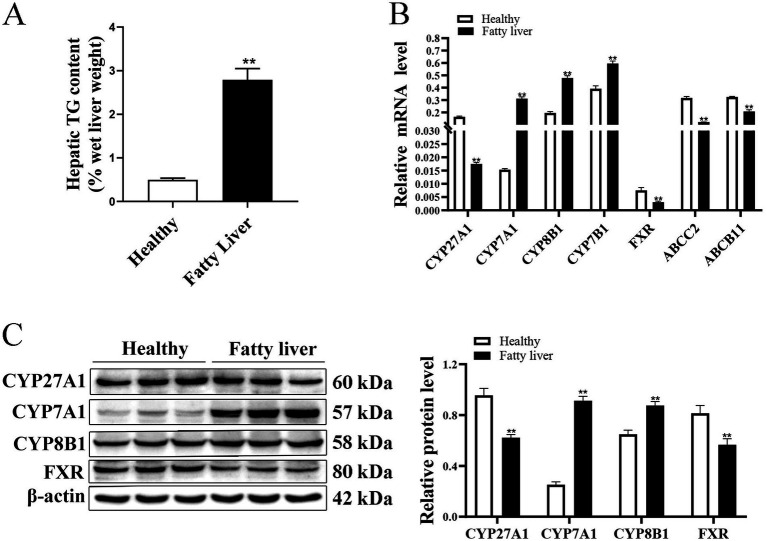
Bile acid metabolism-related factors in healthy or fatty liver cows. Liver tissue from healthy cows (*n* = 6) and cows with fatty liver (*n* = 6) was biopsied and used for mRNA and protein abundance. **(A)** Liver TAG content. **(B)** Relative mRNA expression of *CYP7A1, CYP8B1, CYP27A1, CYP7B1, FXR, ABCC2,* and *ABCB11* was calculated using the 2^−ΔΔCT^ method with *GAPDH* and *ACTB* as reference genes. **(C)** Representative Western blots of *CYP7A1*, *CYP8B1*, *CYP27A1*, and *FXR* from one experiment, and protein abundance of *CYP7A1*, *CYP8B1*, *CYP27A1*, and *FXR*. Comparisons among groups were calculated using a two-tailed unpaired Student’s *t*-test. Data reported are means ± SEM, *p ≤ 0.05, **p ≤ 0.01 indicate differences from healthy controls.

The TAG content in fatty liver cows was greater compared to healthy control cows (*p* < 0.01; Figure 1A).

Relative mRNA and protein abundance of BA metabolism-related factors *CYP27A1* and *FXR* and mRNA abundance of *ABCC2* and *ABCB11* were lower in fatty liver cows compared to healthy controls.

In contrast, mRNA and protein abundance of *CYP8B1* and *CYP7A1* and mRNA abundance of *CYP7B1* were greater in fatty liver cows compared to healthy controls (*p* < 0.05; Figures 1B,C).

### 3.2 Effects of FXR activation and inhibition on bile acid metabolism

**Figure 2:**
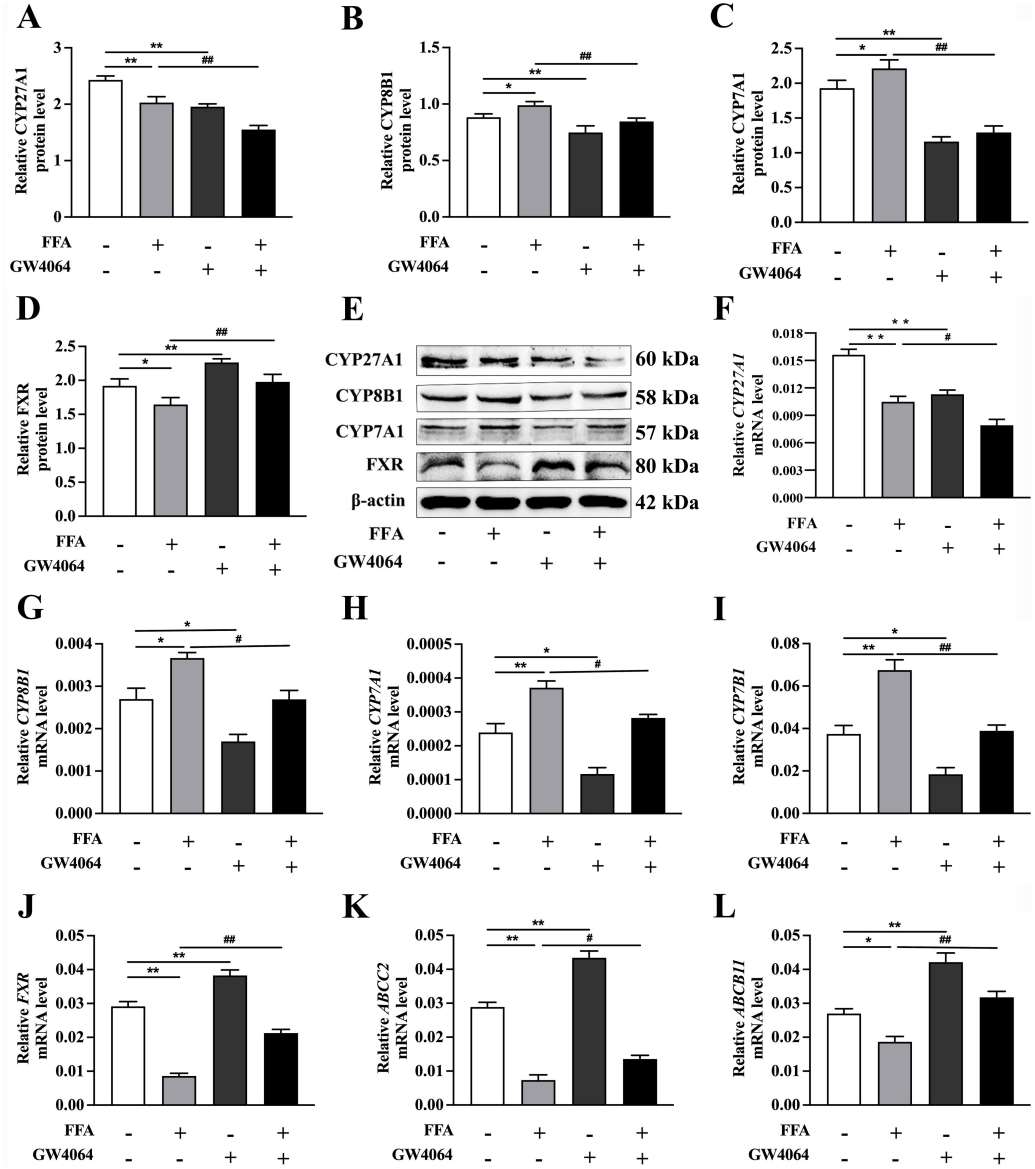
Effects of *FXR* activator GW4064 on bile acid metabolism in calf primary hepatocytes. Calf primary hepatocytes (*n* = 9) were treated with 5 μ*M FXR* activator GW4064 for 12 h, then incubation with or without 1.2 m*M* FFA. **(A–D)** Protein abundance of *CYP27A1*, *CYP8B1*, *CYP7A1*, and *FXR*. **(E)** Representative Western blot analysis of *CYP27A1*, *CYP8B1*, *CYP7A1*, and *FXR*. **(F–L)** Relative mRNA expression of *CYP27A1, CYP8B1, CYP7A1, CYP7B1, FXR, ABCC2*, and *ABCB11* was calculated using the 2^−ΔΔCT^ method with *GAPDH* and *ACTB* as reference genes. Data were analyzed using a one-way ANOVA with subsequent Bonferroni correction. Data reported are means ± SEM.

**Figure 3:**
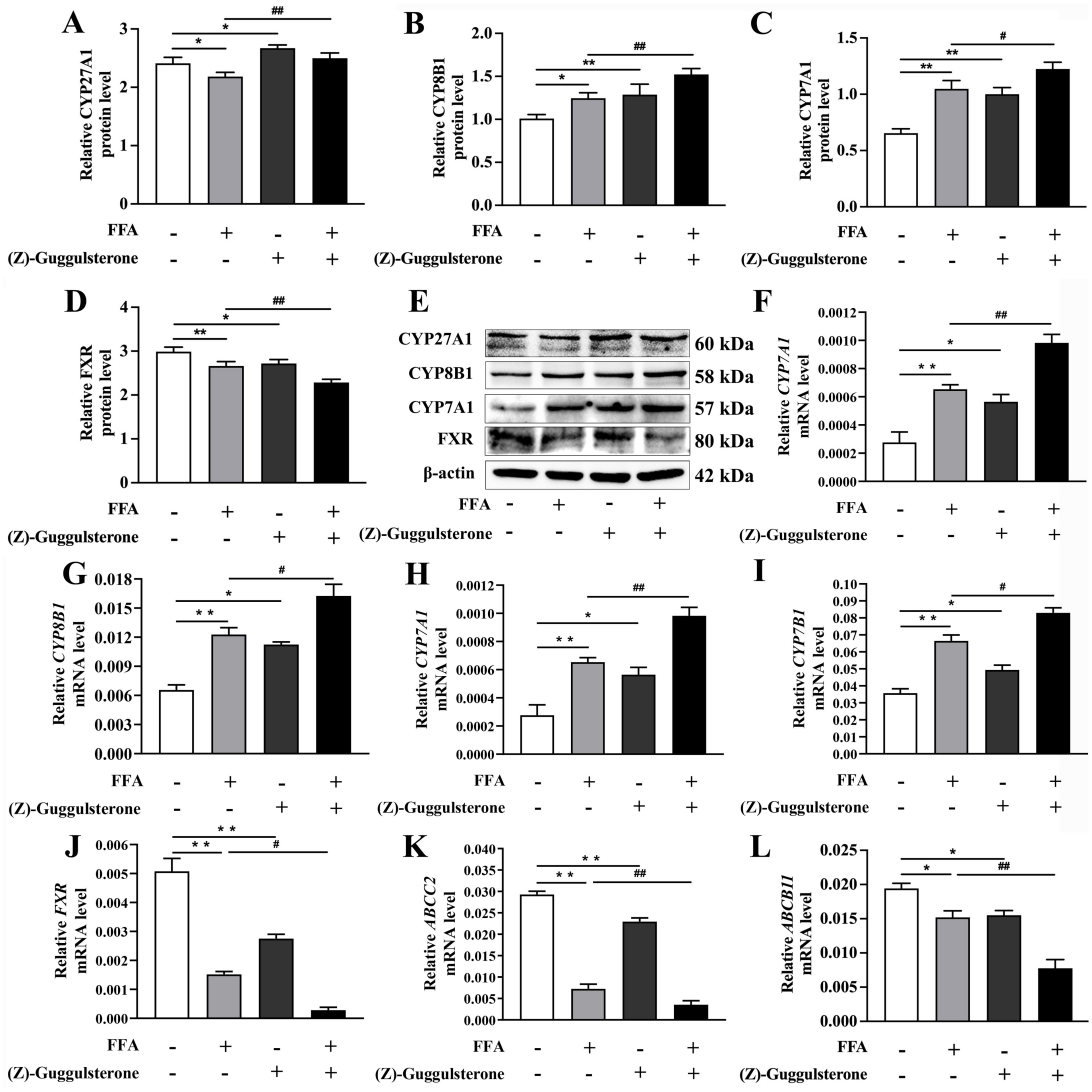
Effects of *FXR* inhibitor (Z)-guggulsterone on bile acid metabolism in calf primary hepatocytes. Calf primary hepatocytes (*n* = 9) were treated with 5 μ*M* *FXR* inhibitor (Z)-guggulsterone for 12 h then incubation with or without 1.2 m*M* FFA. **(A–D)** Protein abundance of *CYP27A1*, *CYP8B1*, *CYP7A1*, and *FXR*. **(E)** Representative Western blot analysis of *CYP27A1*, *CYP8B1*, *CYP7A1*, and *FXR*. **(F–L)** Relative mRNA expression of *CYP27A1, CYP8B1, CYP7A1, CYP7B1, FXR, ABCC2*, and *ABCB11* were calculated using the 2^−ΔΔCT^ method with *GAPDH* and *ACTB* as reference genes. Data were analyzed using a one-way ANOVA with subsequent Bonferroni correction. Data reported are means ± SEM, *p ≤ 0.05, **p ≤ 0.01 indicate differences from control. ^#^*p* ≤ 0.05, ^##^*p* ≤ 0.01 indicate differences from FFA alone.

Compared to the control, relative mRNA and protein abundance of *CYP8B1* and *CYP7A1*, and mRNA abundance of *CYP7B1*, were greater in response to 1.2 m*M* FFA treatment. Conversely, mRNA and protein abundance of *CYP27A1* and *FXR* and mRNA abundance of *ABCC2* and *ABCB11* were lower (*p* < 0.05; Figures 2, 3).

As expected, the *FXR* activator GW4064 led to lower mRNA and protein abundance of *CYP27A1*, *CYP8B1*, and *CYP7A1* and mRNA abundance of *CYP7B1* compared to the controls (*p* < 0.05; Figures 2A–I).

However, the mRNA and protein abundance of *FXR* and the mRNA abundance of *ABCC2* and *ABCB11* were greater in cells treated with GW4064 than controls (*p* < 0.01; Figures 2D, 2J–L).

Compared to the control, hepatocytes treated with the *FXR* inhibitor (Z)-guggulsterone had greater mRNA and protein abundance of *CYP27A1*, *CYP8B1*, and *CYP7A1*, and greater mRNA abundances of *CYP7B1* (*p* < 0.05; Figures 3A–I).

In contrast, mRNA and protein abundance of *FXR* and mRNA abundance of *ABCC2* and *ABCB11* were lower in cells treated with the (Z)-guggulsterone compared to controls (*p* < 0.05; Figures 3D, 3J–L).

Compared to the FFA group, mRNA and protein abundance of *CYP27A1*, *CYP8B1*, and *CYP7A1* and the mRNA abundance of *CYP7B1*, were lower in cells from the GW4064 + FFA group (*p* < 0.05; Figures 2A–I).

In contrast, mRNA abundance of *ABCC2* and *ABCB11* and mRNA and protein abundance of *FXR* were greater in cells with GW4064 + FFA compared to the FFA group (*p* < 0.05; Figures 2D, 2J–L).

Compared to the FFA group, mRNA and protein abundance of *CYP27A1*, *CYP8B1*, and *CYP7A1* and mRNA abundance of *CYP7B1* were greater in cells from the (Z)-guggulsterone + FFA group (*p* < 0.05; Figures 3A–I), while mRNA abundance of *ABCC2*, *ABCB11*, and mRNA and protein abundance of *FXR* in cells with (Z)-guggulsterone + FFA were lower than those in the FFA group (*p* < 0.05; Figures 3D, 3J–L).

### 3.3 Effects of FXR activation and inhibition on lipid synthesis

**Figure 4:**
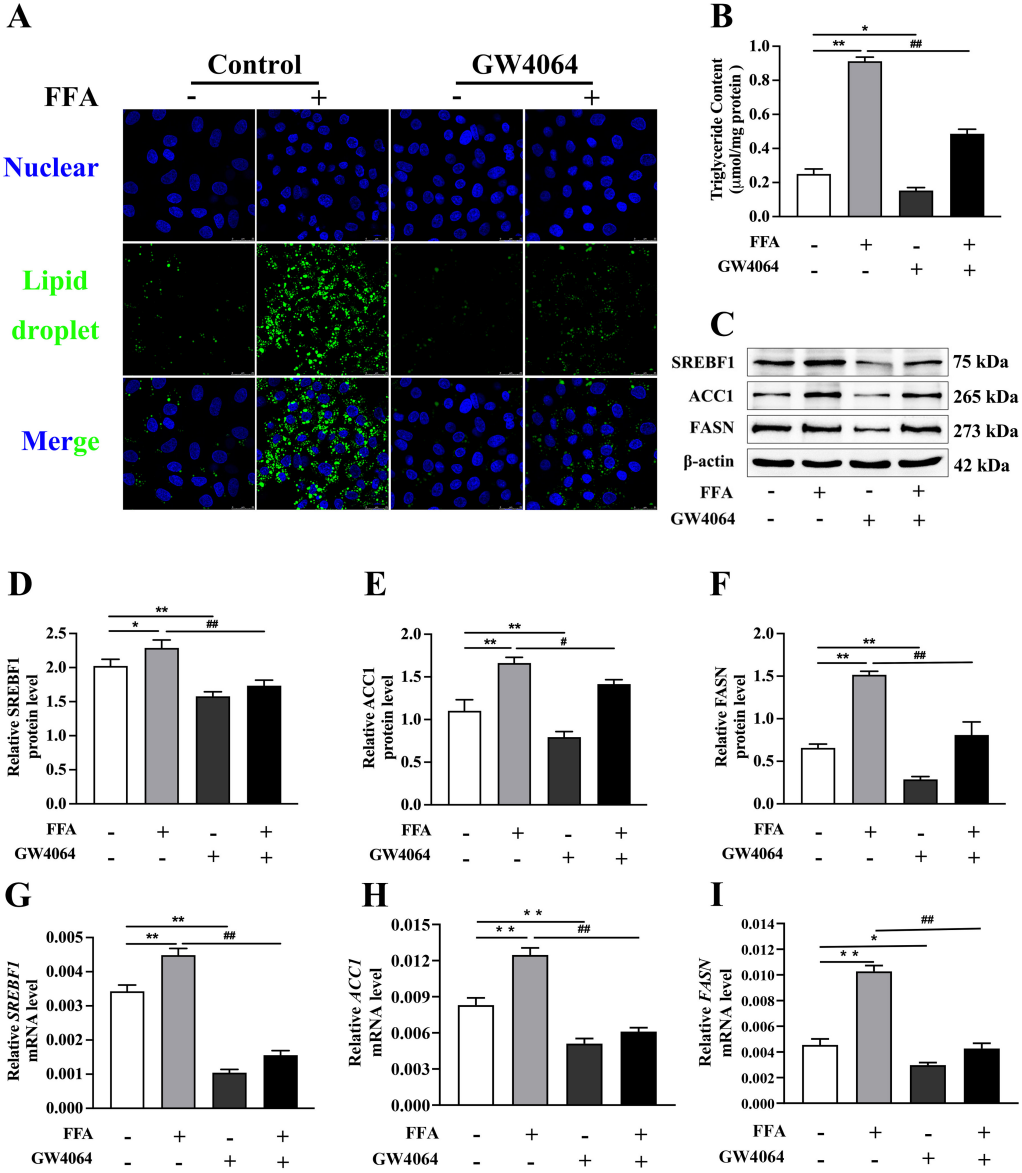
Effects of *FXR* activator GW4064 on lipid synthesis in calf primary hepatocytes. Calf primary hepatocytes (*n* = 9) were treated with 5 μ*M* *FXR* activator GW4064 for 12 h then incubation with or without 1.2 m*M* FFA. **(A)** Lipid droplet fluorescence in calf primary hepatocytes. **(B)** Triglyceride content in hepatocytes. **(C)** Representative Western blot analysis of *SREBF1*, *ACC1*, and *FASN*. **(D–F)** Protein abundance of *SREBF1*, *ACC1*, and *FASN*. **(G–I)** Relative mRNA expression of *SREBF1*, *ACC1*, and *FASN* were calculated using the 2^−ΔΔCT^ method with *GAPDH* and *ACTB* as reference genes. Data were analyzed using a one-way ANOVA with subsequent Bonferroni correction. Data reported are means ± SEM, *p ≤ 0.05, **p ≤ 0.01 indicate differences from control. ^#^*p* ≤ 0.05, ^##^*p* ≤ 0.01 indicate differences from FFA alone.

Compared to the control, the concentration of TAG and accumulation of lipid droplets were greater in hepatocytes challenged with FFA (Figures 4A,B).

However, hepatocytes treated with GW4064 had lower TAG concentration than the control (*p* < 0.05; Figure 4A).

TAG concentration in hepatocytes in the GW4064 + FFA group was lower than that in the FFA group (*p* < 0.01; Figure 4A).

Compared to the control, mRNA and protein abundance of *SREBF1*, *ACC1*, and *FASN* were greater in response to 1.2 m*M* FFA treatment (*p* < 0.05; Figures 4C–I).

Compared to the FFA group, mRNA and protein abundance of *SREBF1*, *ACC1*, and *FASN* were lower in cells with GW4064 + FFA (*p* < 0.05, Figures 4D–I).

Compared to the control, hepatocyte TAG concentration in the (Z)-guggulsterone group was increased.

**Figure 5:**
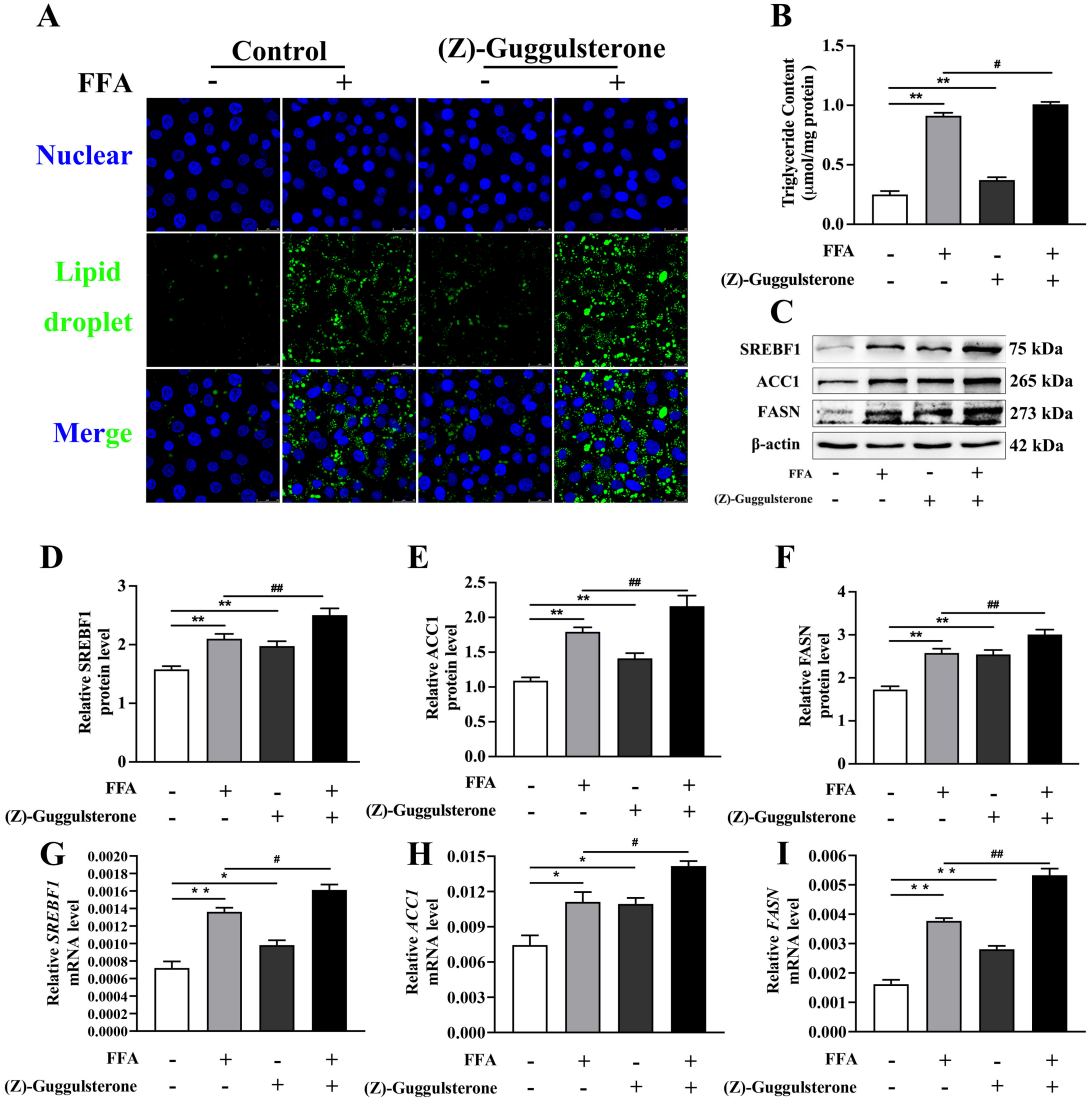
Effects of *FXR* inhibitor (Z)-guggulsterone on lipid synthesis in calf primary hepatocytes. Calf primary hepatocytes (*n* = 9) were treated with 5 μ*M* *FXR* inhibition (Z)-guggulsterone for 12 h then incubation with or without 1.2 m*M* FFA. **(A)** Lipid droplet fluorescence in calf primary hepatocytes. **(B)** Triglyceride content in hepatocytes. **(C)** Representative Western blot analysis of *SREBF1*, *ACC1*, and *FASN*. **(D–F)** Protein abundance of *SREBF1*, *ACC1*, and *FASN*. **(G–I)** Relative mRNA expression of *SREBF1*, *ACC1*, and *FASN* were calculated using the 2^−ΔΔCT^ method with *GAPDH* and *ACTB* as reference genes. Data were analyzed using a one-way ANOVA with subsequent Bonferroni correction. Data reported are means ± SEM, *p ≤ 0.05, **p ≤ 0.01 indicate differences from control. ^#^*p* ≤ 0.05, ^##^*p* ≤ 0.01 indicate differences from FFA alone.

TAG concentration in the (Z)-guggulsterone + FFA group was greater compared to the FFA group (*p* < 0.05; Figures 5A,B). Compared to the FFA group, (Z)-guggulsterone + FFA led to greater mRNA and protein abundance of *SREBF1*, *ACC1*, and *FASN* (*p* < 0.05, Figures 5C–I).

### 3.4 Effects of FXR activation and inhibition on cholesterol synthesis

**Figure 6:**
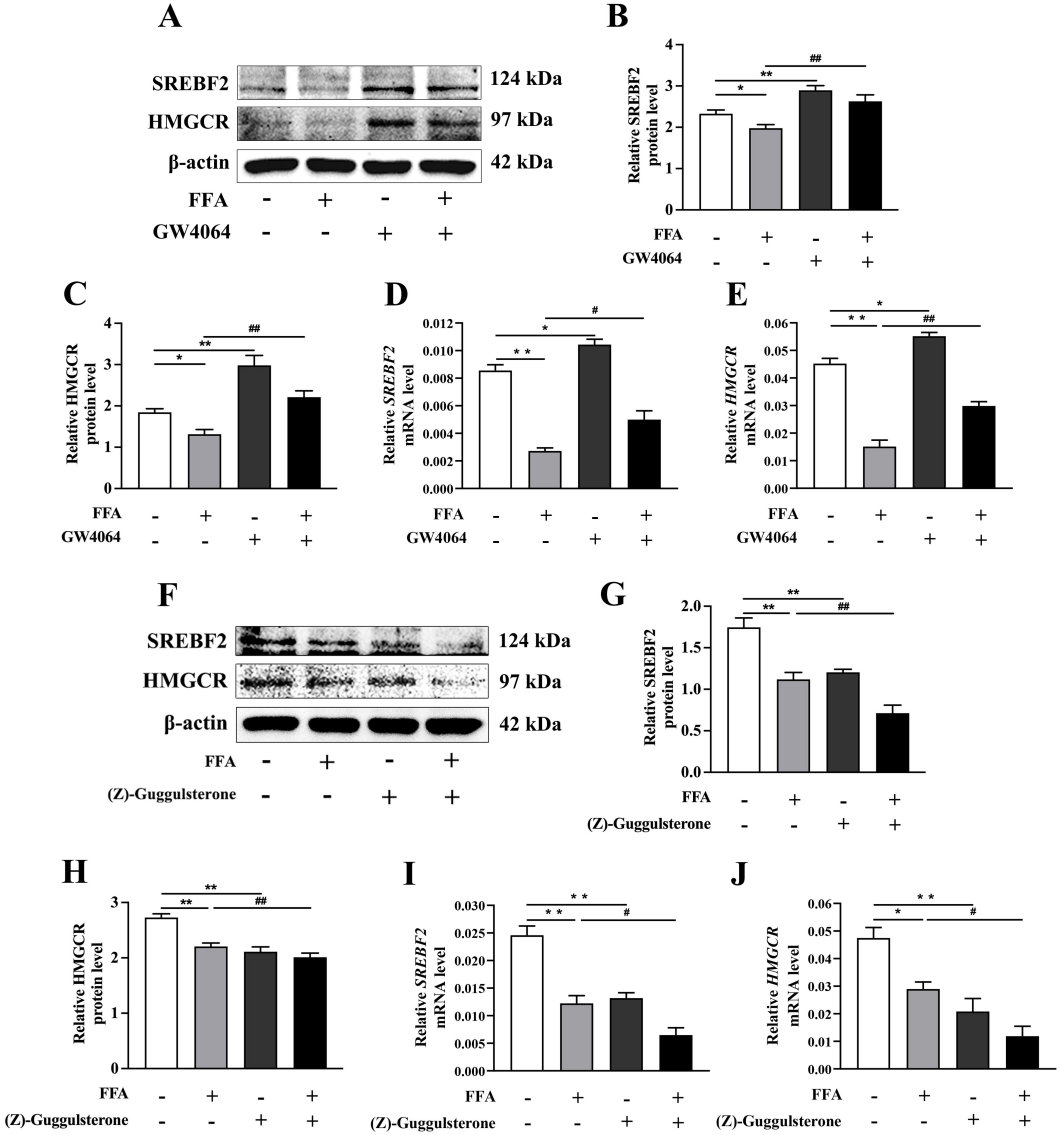
Effects of *FXR* on cholesterol synthesis in calf primary hepatocytes. **(A–E)** Calf primary hepatocytes (*n* = 9) were treated with 5 μ*M* *FXR* activator GW4064 for 12 h then incubation with or without 1.2 m*M* FFA. **(A)** Representative Western blot analysis of *SREBF2* and *HMGCR*. **(B,C)** Protein abundance of *SREBF2* and *HMGCR*. (D, E) Relative mRNA expression of *SREBF2* and *HMGCR* was calculated using the 2^−ΔΔCT^ method with *GAPDH* and *ACTB* as reference genes. **(F–J)** *FXR* inhibition (Z)-guggulsterone for 12 h, then incubation with or without 1.2 m*M* FFA. **(F)** Representative Western blot analysis of *SREBF2* and *HMGCR*. **(G,H)** Protein abundance of *SREBF2* and *HMGCR*. **(I,J)** Relative mRNA expression of *SREBF2* and *HMGCR* were calculated using the 2^−ΔΔCT^ method with *GAPDH* and *ACTB* as reference genes. Data were analyzed using a one-way ANOVA with subsequent Bonferroni correction. Data reported are means ± SEM, *p ≤ 0.05, **p ≤ 0.01 indicate differences from control. ^#^*p* ≤ 0.05, ^##^*p* ≤ 0.01 indicate differences from FFA alone.

Compared to the control, mRNA and protein abundance of *SREBF2* and *HMGCR* were lower in the 1.2 m*M* FFA group (*p* < 0.05; Figure 6).

Interestingly, the *FXR* activator GW4064 led to greater mRNA and protein abundance of *SREBF2* and *HMGCR* than the control (*p* < 0.05; Figures 6A–E).

However, hepatocytes treated with (Z)-guggulsterone had lower mRNA and protein abundance of *SREBF2* and *HMGCR* than control (*p* < 0.01; Figures 6F–I).

Compared to the FFA group, mRNA and protein abundance of *SREBF2* and *HMGCR* were greater in the GW4064 + FFA group (*p* < 0.05, Figures 6A–E).

In contrast, mRNA and protein abundance of *SREBF2* and *HMGCR* in hepatocytes treated with (Z)-guggulsterone + FFA were lower than those in the FFA group (*p* < 0.05, Figures 6F–I).

### 3.5 Effects of FXR activation and inhibition on hepatocyte injury

Compared to the control group, the protein abundance of *VDAC1* was greater in response to 1.2 m*M* FFA treatment.

**Figure 7:**
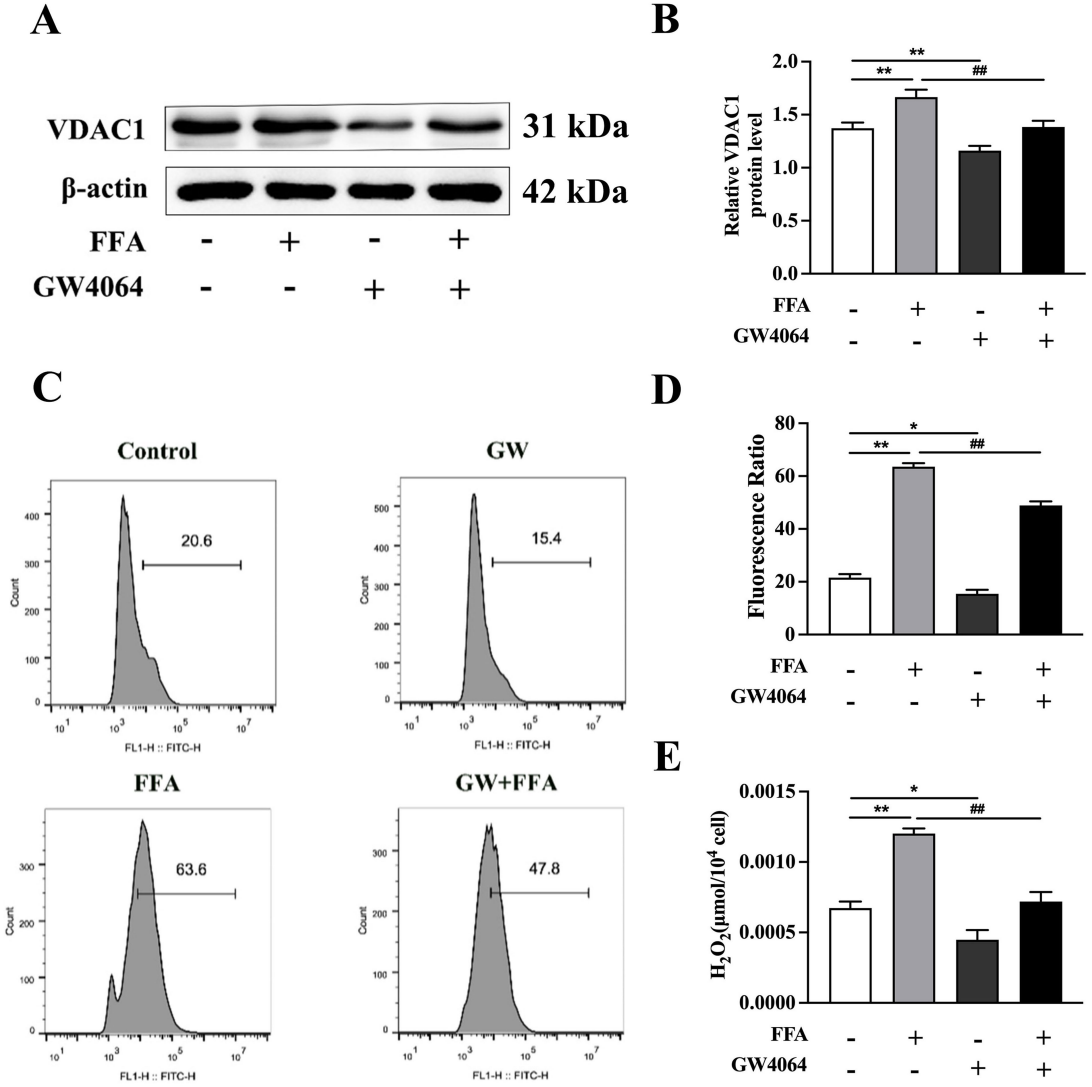
Effects of *FXR* activator GW4064 on cholesterol synthesis in calf primary hepatocytes. Calf primary hepatocytes (*n* = 9) were treated with 5 μ*M* *FXR* activator GW4064 for 12 h, then incubation with or without 1.2 m*M* FFA. **(A)** Representative Western blot analysis of *VDAC1*. **(B)** Protein abundance of *VDAC1*. **(C)** Results of reactive oxygen species (ROS) flow cytometry. **(D)** ROS content in hepatocytes. **(E)** H_2_O_2_ content in hepatocytes. Data were analyzed using a one-way ANOVA with subsequent Bonferroni correction. Data reported are means ± SEM, *p ≤ 0.05, **p ≤ 0.01 indicate differences from control. ^#^*p* ≤ 0.05, ^##^*p* ≤ 0.01 indicate differences from FFA alone.

However, GW4064 led to lower protein abundance of *VDAC1* compared to the control group (*p* < 0.01; Figure 7B).

**Figure 8:**
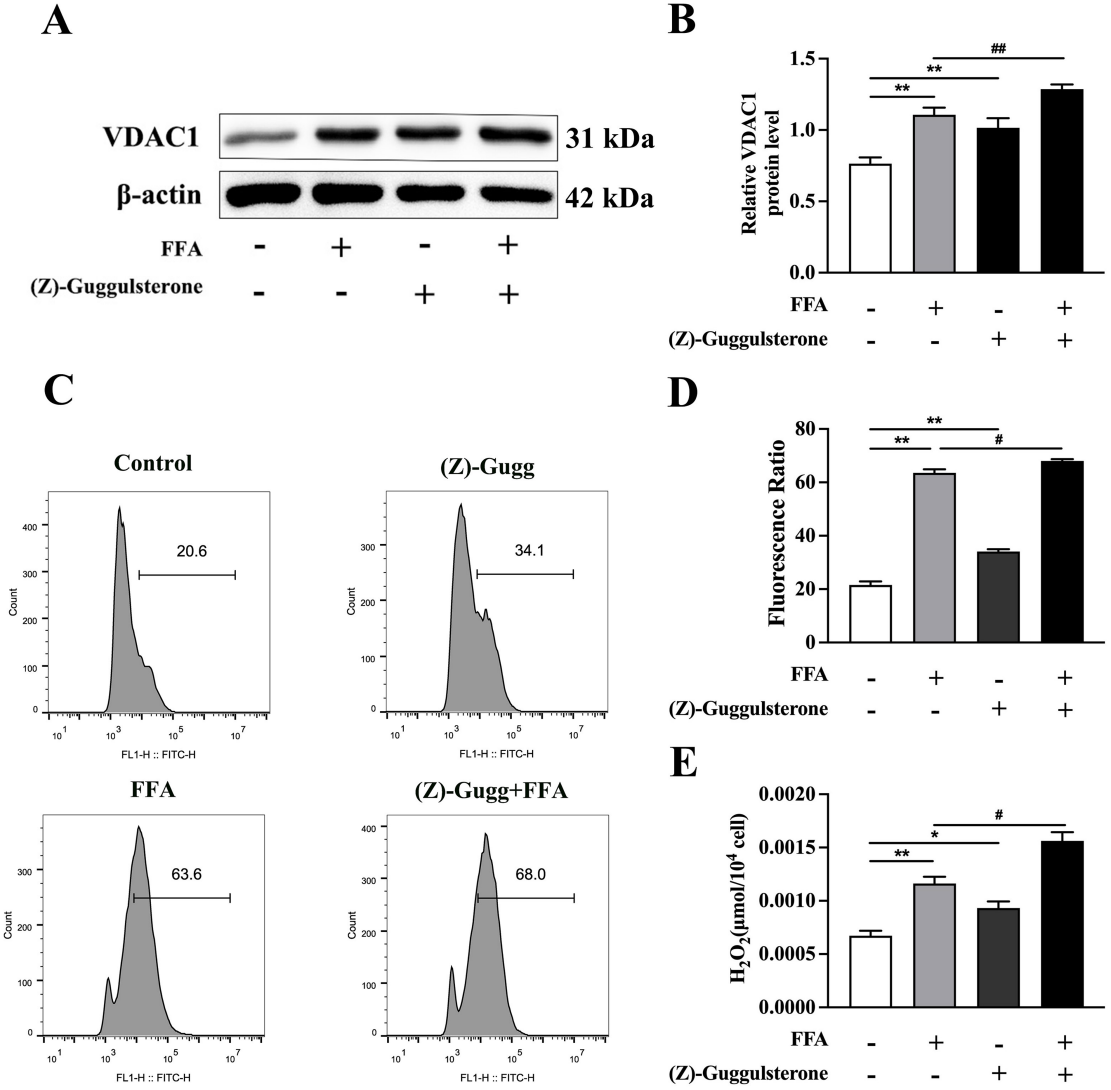
Effects of *FXR* inhibitor (Z)-guggulsterone on cholesterol synthesis in calf primary hepatocytes. Calf primary hepatocytes (*n* = 9) were treated with 5 μ*M* *FXR* inhibitor (Z)-guggulsterone for 12 h, then incubation with or without 1.2 m*M* FFA. **(A)** Representative Western blot analysis of *VDAC1*. **(B)** Protein abundance of *VDAC1*. **(C)** Results of reactive oxygen species (ROS) flow cytometry. **(D)** ROS content in hepatocytes. **(E)** H_2_O_2_ content in hepatocytes. Data were analyzed using a one-way ANOVA with subsequent Bonferroni correction. Data reported are means ± SEM, *p ≤ 0.05, **p ≤ 0.01 indicate differences from control. ^#^*p* ≤ 0.05, ^##^*p* ≤ 0.01 indicate differences from FFA alone.

In contrast, the (Z)-guggulsterone group had greater protein abundance of *VDAC1* than the control (*p* < 0.01; Figure 8B).

Compared to the FFA group, GW4064 + FFA led to lower protein abundance of *VDAC1* (*p* < 0.01, Figure 7B). In contrast, (Z)-guggulsterone + FFA led to greater protein abundance of *VDAC1* compared to the FFA group (*p* < 0.01; Figure 8B).

Compared to the control, ROS and H_2_O_2_ content in hepatocytes were greater in response to 1.2 m*M* FFA treatment (*p* < 0.01; Figures 7, 8).

However, ROS and H_2_O_2_ content in GW4064 were lower compared to the control (*p* < 0.05; Figures 7D,E).

However, ROS and H_2_O_2_ content in the (Z)-guggulsterone groups were greater compared to the control (*p* < 0.01; Figures 8D,E).

In addition, compared to the FFA group, ROS and H_2_O_2_ content were lower in hepatocytes with GW4064 + FFA (*p* < 0.01; Figures 7D,E), whereas ROS and H_2_O_2_ content were greater in hepatocytes with (Z)-guggulsterone + FFA (*p* < 0.05; Figures 8D,E).

## 4 Discussion

Peripartal dairy cows experience severe NEB, resulting in elevated circulating FFA levels that exceed the liver’s capacity for lipid oxidation and secretion. This can result in fatty liver due to the accumulation of TAG (8).

Previous research indicates a functional crosstalk between the BA-dependent *FXR* pathway and intrahepatic TAG metabolism. In human studies, reduced protein levels of *FXR* in nonalcoholic steatohepatitis (NASH) patients compared to non-alcoholic fatty liver disease (NAFLD) patients suggest a protective role for *FXR* in the progression of NAFLD to non-alcoholic steatohepatitis (NASH) (26).

Our investigation revealed that exogenous FFA challenge decreased *FXR* expression in calf hepatocytes. Notably, the *FXR* activator GW4064 reduced TAG accumulation induced by high FFA levels in these cells.

In addition, this study demonstrated that activated *FXR* inhibited the expression of lipid synthesis-related factors *SREBF1*, *ACC1*, and *FASN* in calf hepatocytes. In hypertriglyceridemic mouse models, BA administration downregulated *SREBF1* and consequently reduced plasma TAG levels (27).

Activation of *FXR* has been shown to inhibit *SREBF1*-mediated lipogenesis through the *FXR*–SHP pathway (27). Moreover, previous studies have shown that *FXR* activation reduces FFA levels in both wild-type and diabetic db/db mice (28).

Consistent with these findings, our study demonstrated that the inhibition of *FXR* by (Z)-guggulsterone increased the protein and mRNA abundance of *SREBF1*, as well as TAG content in calf hepatocytes.

Therefore, activating *FXR* can attenuate high FFA-induced TAG levels and lipid droplet accumulation by upregulating *FXR* expression and inhibiting *SREBF1*-mediated hepatic lipogenesis.

*FXR* exerts feedback inhibition on BA synthesis via two primary mechanisms. First, *FXR*-mediated activation of SHP represses *CYP7A1* expression, thereby inhibiting BA synthesis and uptake. Second, BA activates intestinal *FXR*–*FGF15/19* signaling, which subsequently suppresses hepatic *CYP7A1* expression and induces SHP expression in hepatocytes, leading to the inhibition of *CYP8B1* and *CYP7A1* gene transcription (29).

Related studies have shown that systemic disruption of the *FXR* gene has been linked to aberrant cholesterol and lipid homeostasis and is associated with impaired BA transport and reduced expression of ABCB11 (30). Deficiencies in ABCB11 are a significant cause of cholestasis, resulting in hepatotoxicity, inflammation, and oxidative stress within hepatocytes (13, 31).

Our study found that hepatocytes treated with the *FXR* activator GW4064 exhibited reduced *CYP7A1* expression and increased *ABCB11* expression.

Impaired bile secretion can lead to cholestasis, causing the accumulation of bile salts and other toxic substances within hepatocytes. This study found that the expression of BA synthesis-related genes (*CYP7A1*, *CYP8B1*, *CYP27A1*, *CYP7B1*) was downregulated, and BA transporter genes (*ABCC2*, *ABCB11*) were upregulated after the activation of *FXR* by GW4064.

The *FXR* inhibitor (Z)-guggulsterone induced the converse effects. Consistently, previous studies have shown increased hepatic BA accumulation in dairy cows with fatty liver (32).

Therefore, the reduction of hepatic BA accumulation by activating hepatic *FXR* prevents the further development of fatty liver disease caused by intrahepatic cholestasis.

Hepatic cholesterol homeostasis is intricately linked to a cascade of enzymatic reactions involved in BA biotransformation. *FXR* promotes the expression of the scavenger receptor class B type I (SR-B1), which mediates hepatic uptake of cholesteryl esters from high-density lipoprotein (HDL), thereby contributing to reduced plasma HDL levels (14, 33).

Our findings indicate that high FFA levels lead to a decrease in the expression of *SREBF2,* which in turn reduces cholesterol synthesis (9). Actually, cholesterol stimulates *CYP7A1* transcription, and as the rate-limiting enzyme in cholesterol conversion to bile acids, reduced *CYP7A1* expression results in elevated intracellular cholesterol levels (34).

Consistently, *FXR* activator GW4064 led to an upregulated expression of *HMGCR* and *SREBF2*. Our previous studies have indicated that cholesterol availability can promote VLDL excretion and reduce lipid accumulation and oxidative stress in hepatocytes challenged with high concentrations of fatty acids (9).

Therefore, the activated *FXR* was suggested to reduce CYP7A1-induced hepatocytes to synthesize primary bile acids from cholesterol, while promoting cholesterol-induced VLDL excretion in hepatocytes challenged with high concentrations of fatty acids. Furthermore, *FXR* activation has demonstrated protective effects against cellular oxidative stress and mitochondrial damage (35). Moreover, a previous study on BA profiles in periparturient dairy cows indicated that serum concentrations of CA, CDCA, glycocholic acid (GCA), taurocholic acid, glycochenodeoxycholic acid, deoxycholic acid, lithocholic acid (LCA), and glycodeoxycholic acid were lower in high body condition score (BCS) cows compared with normal BCS cows. Conversely, serum concentrations of *β*-muricholic acid (β-MCA) were higher in high BCS cows compared with normal BCS cows (36).

Notably, CA, LCA, CDCA, and GCA are natural agonists of *FXR*, whereas *β*-MCA acts as an antagonist (37).

Importantly, supplementation with BAs during the transition period has been shown to reduce oxidative stress and inflammation, while potentially enhancing the health and lactation performance of dairy cows (38).

Thus, our data suggest that activation of FXR can reduce the synthesis and excretion of BAs and alleviate the lipid accumulation in hepatocytes caused by a high fatty acid load. Furthermore, supplementation with natural agonists bile salt of *FXR,* suggest a beneficial effects on fatty liver in dairy cows.

Although calf hepatocytes were widely used to elucidate regulatory mechanisms of hepatic lipid metabolism in adult ruminants (39, 40). Caution should be exercised when attempting to infer regulatory mechanisms from hepatocytes of 1-day-old calves. Because there are clear differences in aspects of lipid metabolism as a function of age (41). Although the present *in vitro* data suggested that *FXR* affects BA metabolism, the actual role of *FXR* in dairy cow hepatic lipid metabolism remains to be determined.

## 5 Conclusion

A high fatty acid load induces lipid synthesis and BA synthesis, while suppressing cholesterol synthesis and *FXR* expression in primary calf hepatocytes. The activation of *FXR* by GW4064 effectively alleviated high FFA-mediated BA and lipid accumulation in calf hepatocytes, ultimately mitigating cellular oxidative stress. Collectively, this study highlights *FXR*-mediated BA metabolism as a potential therapeutic strategy for reducing FFA-induced lipid accumulation in the liver of dairy cows during the transition period.

## Data Availability

The original contributions presented in the study are included in the article/supplementary material, and further inquiries can be directed to the corresponding authors.
